# Attitudinal Beliefs About Suicidal Behavior and Attitudes Towards Suicide Attempts in Colombian Healthcare Professionals

**DOI:** 10.3390/healthcare12212169

**Published:** 2024-10-31

**Authors:** Marly Johana Bahamón, José Julián Javela, Andrea Ortega Bechara, Andrés Cabezas-Corcione, Lorena Cudris-Torres

**Affiliations:** 1 Faculty of Health Sciences, Research Group I-Flor, Universidad del Sinú, Montería 230001, Colombia; jjavela@gmail.com (J.J.J.); a.ortega.bechara@unisinu.edu.co (A.O.B.); corcioneandres1@gmail.com (A.C.-C.); 2 Department of Social Sciences, Universidad de la Costa, Barranquilla 080002, Colombia; lcudris3@cuc.edu.co

**Keywords:** perceptions toward suicide, attitudes toward suicide, suicide attempt, healthcare professionals, suicide

## Abstract

**Background**: Suicide remains a major global public health concern, ranking among the leading causes of death worldwide. Healthcare professionals play a crucial role in preventing suicide, yet their attitudes and beliefs about suicidal behavior can significantly impact the quality of care provided to individuals at risk. **Objective**: This study aimed to analyze the attitudinal beliefs of Colombian healthcare professionals regarding suicidal behavior and their attitudes toward suicide attempts. **Methods**: A cross-sectional study was conducted with 354 healthcare professionals, including interns, from fields such as medicine, nursing, and nursing assistance. Participants completed the Attitudinal Beliefs Questionnaire about Suicidal Behavior (CCCS-18) and the Attitudes Towards Suicide Attempt Scale (ASETSA). Descriptive and inferential statistical analyses were performed to examine the relationship between professional experience and attitudes toward suicidal behavior. **Results**: The findings revealed that many participants exhibit attitudes that legitimize suicide in cases of extreme suffering, such as in terminal illness. However, there were significant gaps in comprehensive approaches to suicide prevention, with varying levels of commitment to individual and institutional support. Clinical experience was identified as a key factor in shaping these attitudes, with more experienced professionals showing a nuanced understanding of suicide triggers and prevention. **Conclusions**: The results underscore the importance of ongoing training programs that not only improve technical knowledge but also address misconceptions and stigmas related to suicide. These findings have direct implications for developing targeted interventions and educational programs within the Colombian healthcare context to ensure more compassionate and effective care for individuals at risk of suicide.

## 1. Introduction

Suicide is a critical public health issue affecting millions globally, consistently ranking among the leading causes of death worldwide [1,2]. This phenomenon not only has a devastating emotional and psychological impact on families and communities but also presents significant challenges to healthcare systems and the professionals within them, who play a pivotal role in suicide prevention [3].

A review of the literature highlights that suicide is one of the most preventable causes of death, with primary care physicians being crucial to prevention efforts. It is reported that 45% of individuals who die by suicide have had contact with a primary care physician within 30 days prior to their death, emphasizing the critical opportunity for timely intervention [4].

### 1.1. Recent Research on Beliefs About Suicide Among Healthcare Professionals

The attitudes and beliefs held by healthcare professionals toward suicide significantly influence how they interact with at-risk patients, potentially affecting the quality of care provided [5,6]. Thus, exploring healthcare professionals’ beliefs and attitudes toward suicide is increasingly important due to the need to improve care for individuals at risk. Misconceptions and stigmas related to suicide can result in inadequate case management, potentially increasing the risk of future suicide attempts. Recent research indicates that myths about suicide are prevalent among first responders and can negatively impact the quality of initial care [7]. These misconceptions may also be present among healthcare professionals, potentially affecting their ability to provide appropriate care [8,9].

Despite efforts to train healthcare professionals in identifying and managing suicidal behavior, significant barriers continue to hinder the provision of effective care. A well-documented issue is the widespread lack of confidence and knowledge among healthcare professionals when dealing with suicidal patients. Several studies have found that many professionals are not adequately informed about suicide risk factors and prevention strategies, which negatively impacts their attitudes toward at-risk patients [10,11,12]. Inadequate preparation can result in poor management and an increased risk of suicide [13].

The literature reveals significant variability in healthcare professionals’ knowledge and attitudes toward suicide. For instance, a systematic review identified substantial gaps in suicide-related knowledge, particularly among professionals in primary care settings. This finding highlights the need for implementing training programs that boost confidence and enhance the ability of professionals to provide effective support to individuals at risk of suicide [10].

Other studies have evaluated the attitudes and beliefs of healthcare professionals toward suicide in various contexts. For example, a study conducted in a public hospital in Lima, Peru, using the Attitudinal Beliefs Questionnaire about Suicidal Behavior (CCCS-18), found that most professionals held neutral attitudes and beliefs toward suicide. However, more favorable attitudes toward suicide were observed in the contexts of terminal illness and morality, emphasizing the need for continuous education and access to support services to improve attitudes and the clinical management of suicidal behavior [5].

Attitudes toward suicide vary significantly across different groups of healthcare professionals. Schulz et al., 2024 [14] found that medical students tend to hold more negative attitudes toward suicide compared to practicing physicians, emphasizing the need for earlier and continuous training in suicide intervention. Such training not only improves attitudes toward suicidal patients but also has the potential to reduce suicide rates by enhancing the quality of care.

Among mental health care providers, research has shown that those who have experienced a patient’s suicide report improved skills but also express the need for more in-depth training on the subject. This underscores the importance of providing ongoing, high-quality training to build confidence and competence in suicide prevention [15].

### 1.2. Knowledge and Training in Suicide Prevention Among Healthcare Professionals

In the United States, Washington became the first state to mandate that healthcare professionals complete training on suicide management, which led to significant improvements in participants’ knowledge, attitudes, and confidence [16]. In contrast, Shah et al. (2016), in their assessment of training needs in India, found that many primary care professionals lacked adequate competence in suicide risk assessment and management, underscoring the importance of developing tailored training programs based on local needs [17].

Educational interventions have also proven effective in improving healthcare professionals’ attitudes toward suicide. For example, a suicide prevention training program evaluated by Faria et al., 2022 [18] in Brazil demonstrated that participants in the intervention group developed more positive attitudes and a greater perceived ability to manage high-risk situations. These findings suggest that continuous education plays a crucial role in shaping attitudes and enhancing clinical practices related to suicide.

### 1.3. Emotional and Professional Impact of Suicide on Healthcare Personnel

Another area of research focuses on the emotional and professional impact of patient suicide on healthcare professionals, which can be significant. Jupina et al., 2024 [19] found that 51% of healthcare professionals have experienced a patient suicide, resulting in changes to their clinical practices and negatively affecting their emotional well-being. This highlights the need for support interventions to help professionals process these experiences and maintain their personal and professional well-being [20].

Similarly, several studies have explored the risk and protective factors related to suicide among healthcare professionals [15,17,21]. Bhatia et al., 2023, identified professional burnout and access to lethal means as key risk factors for suicide among physicians. In contrast, strong support networks and wellness programs act as protective factors, helping to mitigate some of the risks associated with working in the medical field [22].

### 1.4. Suicide Education and Training for Healthcare Professionals

A comprehensive review by Mann et al. (2005) concluded that physician education and restricting access to lethal means are key strategies for suicide prevention [23]. However, other strategies, such as public education and screening programs, require further evidence to determine their effectiveness. In a subsequent study, Ferguson et al. (2019) examined a suicide prevention education program in rural Australia and found significant improvements in the attitudes and confidence of health and human service professionals, highlighting the positive impact of brief training programs [24].

Simulation training has also emerged as a promising tool for educating healthcare professionals in suicide prevention. A meta-analysis by Richard et al. (2023) on the use of simulation in training showed significant improvements in attitudes, skills, and knowledge among healthcare workers. Despite these positive outcomes, evidence of direct benefits to patients remains limited, requiring further research to assess its broader impact [25].

Solin et al. (2021) demonstrated that a brief, three-hour educational intervention was effective in increasing the self-perceived competence of primary care professionals in assessing and managing suicide risk. Although improvements were significant across all professional groups, the study emphasized the need for continued follow-up training to maintain these gains. Similarly, Roos et al. (2019) investigated gaps in healthcare provision prior to suicides in Sweden and identified “suicide risk assessment” and “treatment” as critical areas. However, the proposed actions, such as continuing education, did not always align with the actual needs identified [26,27].

Mitchell et al. (2020) analyzed how appropriate training can reduce negative attitudes and anxiety among mental health professionals toward suicidal patients [28]. Their findings indicate that effective training enhances self-efficacy in assessing suicide risk and reduces distress and avoidance behaviors. Likewise, Muehlenkamp et al. (2023) found that emergency nurses who received formal training in suicide intervention exhibited greater confidence and lower levels of burnout compared to those who did not receive adequate training [21].

Despite advancements in research on healthcare professionals’ attitudes and beliefs toward suicide, there are areas that require further attention. While recent studies have examined the impact of training and beliefs on the care of suicidal patients [15,21,28], most of the research has focused on specific geographic contexts, such as the United States and Europe [16,25], and primarily on physicians. However, there is a notable lack of studies exploring the attitudes and beliefs of different groups of healthcare professionals in regions like Latin America (Yuncar-Fajardo, et al., 2023), particularly in Colombia [5]. Differences in training, cultural context, and available resources may significantly influence perceptions and clinical practices related to suicide management [17].

Furthermore, although there is growing evidence that stigmatizing beliefs and myths about suicide negatively impact the quality of care, there is still a need for a systematic evaluation of how these factors affect various levels of healthcare personnel [4]. This includes not only physicians but also medical residents, nurses, and nursing assistants, all of whom play a crucial role in patient care and the early detection of suicide risk. While some studies have reported improvements in attitudes through training, the long-term impact of these interventions on the clinical practice of these groups, especially in low- and middle-income countries, has not been sufficiently investigated [21,25,28].

This gap in the literature highlights the need for more research that comparatively examines the attitudes, beliefs, and preparedness of different levels of healthcare professionals across diverse cultural contexts [11,16]. Our study, which includes physicians, medical residents, nurses, and nursing assistants in Colombia, seeks to address this gap by providing a focused perspective on a Latin American context that has been underexplored.

Considering the above, this study aims to examine attitudes toward suicide attempts and attitudinal beliefs about suicidal behavior among different groups of healthcare professionals in Colombia, including physicians, medical residents, nurses, and nursing assistants. It is proposed that these attitudes may vary significantly depending on the professional area and both professional and non-professional experience with suicidal patients. Specifically, it is hypothesized that healthcare professionals with direct experience managing patients who have attempted suicide will develop more favorable and less stigmatizing attitudes toward these behaviors. Furthermore, non-professional experiences, such as having a friend or family member who has attempted suicide, are also expected to influence professionals’ attitudes and beliefs. Therefore, this study seeks not only to identify these differences but also to provide valuable insights to inform recommendations aimed at improving training and support for healthcare professionals, with the goal of optimizing care for individuals at risk of suicide in the Colombian context.

## 2. Method

### 2.1. Participants and Procedure

A total of n = 354 healthcare professionals and intern professionals in the same field with clinical practice experience participated, of whom n = 153 (43.2%) were men and n = 201 (56.7%) were women, aged between 18 and 76 years (M = 32.7, SD = 11.76). According to profession, the age distribution was as follows: physicians between 21 and 63 years (M = 36.3, SD = 11.2), medical interns between 18 and 26 years (M = 22, SD = 2.0), nursing professionals between 19 and 62 years (M = 35.6, SD = 10.4), nursing interns between 18 and 32 years (M = 21.1, SD = 3.0), and nursing assistants between 18 and 76 years (M = 35.2, SD = 12.5).

The recruitment of participants was conducted in hospitals and health centers selected from the official registry of the Ministry of Health and Social Protection of Colombia, specifically from the Registry of Healthcare Providers and Service Sites corresponding to a major city in the country. From this registry, a total of 50 public and private institutions registered with the city’s Health Secretariat were identified, which had coverage exceeding 200,000 users. Of these, 16 institutions were selected, as they had medium to high complexity levels of care and frequently handled suicide attempt cases in their emergency services. The selection of these institutions was based on the need to ensure that the recruited professionals had exposure to the care of patients in crisis situations, which aligned with the objectives of the study.

In this study, healthcare professionals and medical and nursing interns over the age of 18, with recent clinical experience, who belonged to the fields of medicine, nursing, or nursing assistance, and who were able to read and understand Spanish to complete the questionnaires were included. The inclusion criteria also considered the participants’ willingness to voluntarily complete the instruments. On the other hand, those who did not provide informed consent, those without recent practical clinical experience, those who did not complete the questionnaires properly, and those experiencing an acute psychological crisis that could affect their responses were excluded. These criteria ensured an appropriate and relevant sample for the study’s objectives.

The recruitment process was initially carried out through the human resources department of each institution, where professionals were invited to participate in the study. Subsequently, a convenient time was agreed upon by phone for each participant. At the available time for each professional, a senior research assistant conducted the individual administration of the booklet containing the evaluation scales and handled the informed consent process.

The instructions were uniform for all participants, and it was requested that the assessment be conducted in a quiet space, generally at the beginning or end of the participant’s workday, to minimize distractions. The process was monitored by the responsible assistants, who were trained to resolve any questions during the administration of the instruments. Additionally, participants were provided with the contact information of the study coordinators for any further inquiries.

The approximate time to complete the questionnaires was 20 to 30 min, and the data collection process extended over a period of six months.

### 2.2. Instruments

Attitudinal Beliefs about Suicidal Behavior Questionnaire or CCCS-18 [29]: This instrument was designed in Spanish and validated with a Spanish university population. It consisted of 18 items with a 7-point Likert-type scale ranging from 1 (strongly disagree) to 7 (strongly agree). It evaluated four factors: I. Legitimization of Suicide, which included 6 items (1, 5, 8, 10, 14, and 18) related to the view of suicide as something rationally acceptable; II. Suicide in Terminally Ill Patients, which included 4 items (2, 6, 11, and 15) related to suicide in patients with no possibility of survival; III. Moral Dimension of Suicide, composed of 4 items (3, 7, 12, and 16) that were reverse scored; IV. Own Suicide, composed of 4 items (4, 9, 13, and 17), indicating a view of suicidal behavior as an escape from a particular situation. The reliability indices of the instrument reported by its authors ranged from α = 0.73 to 0.84 for each factor (Legitimization of Suicide α = 0.84, Suicide in Terminally Ill Patients α = 0.82, Moral Dimension of Suicide α = 0.78, Own Suicide α = 0.73). Additionally, they reported a reliability index for the entire scale of α = 0.87. For this study, the reliability of the instrument was analyzed among the participants, who were Colombian healthcare professionals. The reliability indices for each factor ranged between α = 0.32 and 0.79 (Legitimization of Suicide α = 0.56, Suicide in Terminally Ill Patients α = 0.79, Moral Dimension of Suicide α = 0.64, Own Suicide α = 0.33), with an overall reliability index of α = 0.62. The validity of this scale was tested using exploratory factor analysis, identifying that the four factors explained 60.74% of the variance. Furthermore, through discriminant validity analysis, significant differences (*p* = <0.05 and *p* = <0.01) were identified with suicidal ideation, suicide attempts, and suicide probability.

Attitude Scale Towards Attempted Suicide Cases (ASETSA) [9]: The ASETSA scale was developed to evaluate the attitudes of emergency medical teams toward suicide attempt cases. The instrument measured six factors: I. Prevention and Protection (items 1, 2, 3, 4, 5, 6); II. Individual Help (items 6, 7, 8); III. Institutional Help (items 9 and 10); IV. Triggers and Psychopathology (items 11, 12, 13, 14, 15, 16, 17); V. Causal Attributions (items 18, 19, 20, 21, 22, 23); and VI. Medical Help (24, 25, 26, 27, 28), explaining 58.5% of the total variance. It had high internal consistency reflected by a Cronbach’s alpha of α = 0.84 and a test-retest reliability of 0.70. For this study, a reliability analysis was conducted with Colombian healthcare professionals, identifying an overall internal consistency of α = 0.85. The consistency for each factor was as follows: Factor I. Prevention and Protection (α = 0.70); Factor II. Individual Help (α = 0.60); Factor III. Institutional Help (α = 0.76); Factor IV. Triggers and Psychopathology (α = 0.66); Factor V. Causal Attributions (α = 0.71); and Factor VI. Medical Help (α = 0.80). Content validity was assessed using an index with a cutoff point of 0.60, and structural validity was confirmed through exploratory factor analysis.

The two instruments applied were previously analyzed to ensure their cultural adaptation and understanding. Prior to the implementation of the study, a pilot test was conducted with 12 university students in medicine, nursing, and nursing assistance to evaluate the use and understanding of the questionnaires (CCCS-18 and ASETSA). This pilot test confirmed the feasibility of the scales in the Colombian context, ensuring they were properly interpreted by healthcare professionals.

### 2.3. Statistical Analysis

Descriptive statistics (means, standard deviations, and percentages) were applied to analyze the sociodemographic characteristics and the dimensions of attitudinal beliefs about suicidal behavior and attitudes toward suicide attempts. The Shapiro–Wilk test was used to test the assumption of data normality, considering that the sample size was intermediate, as this test tends to be more sensitive to deviations from normality than the Kolmogorov–Smirnov test [30].

Since the data showed a non-normal distribution, the Kruskal–Wallis test was used to evaluate differences between groups based on profession as a fixed factor and each dimension of the applied scales as dependent variables. Subsequently, Dunn’s post hoc test was applied to identify specific differences between professional groups.

Additionally, the Mann–Whitney U test was used to compare independent samples based on professional experience with suicidal patients and non-professional experience with suicidal individuals. The effect size was calculated using rank–biserial correlation.

Data were analyzed using IBM SPSS^®^ 25.0 (IBM Corporation, Armonk, NY, USA) and JASP 0.18.

### 2.4. Ethics Committee

The data in this manuscript were collected and processed in accordance with the provisions of the ethics committee of the University of Sinú, Act No. 003, 20 April 2024 code 003-2024.

## 3. Results

A total of n = 354 healthcare professionals and intern professionals in the same field with clinical practice experience participated, of whom n = 153 (43.2%) were men and n = 201 (56.7%) were women, aged between 18 and 76 years (M = 32.7, SD = 11.76). Most participants belonged to a low or lower-middle economic level (48.9%), followed by middle or upper-middle economic levels (44.1%), and high or very high economic levels (7.0%). The distribution of professions was as follows: medicine (22.0%), medical interns (13.8%), nursing (24.9%), nursing interns (7.3%), and nursing assistants (31.9%). Professional experience ranged from 0 to 40 years (M = 7.31 years, SD = 8.7 years). For the study, participants were asked if they had professional experience with suicidal patients, with 27.9% responding affirmatively; additionally, they were asked if they had non-professional experience with individuals exhibiting suicidal behavior (family members, friends, or acquaintances), to which 58.7% responded affirmatively.

Table 1 presents a descriptive analysis of attitudinal beliefs about suicidal behavior (CCS-18) in a sample of 354 individuals, revealing significant differences across various dimensions. In the Legitimization of Suicide, a moderate trend is observed, with a mean of 9.96 and positive skewness (Skewness = 1.67), indicating that some individuals in the sample tend to justify suicide under certain circumstances. Similarly, attitudes toward Suicide in Terminally Ill Patients show a mean of 8.68 and a distribution skewed toward higher extreme values (Skewness = 1.50), suggesting that some individuals view suicide as an option in situations of extreme suffering.

On the other hand, the Moral Dimension of Suicide exhibits a strong moral perception, with a mean of 14.66 and a relatively symmetrical distribution (Skewness = 0.22). This trend suggests that most participants consider suicide from an ethical or moral perspective. Regarding the perception of one’s Own Suicide, the mean of 7.83 indicates a more cautious or reserved attitude, reflected in lower positive skewness (Skewness = 0.82). Normality tests show statistical significance (*p* < 0.001) across all dimensions, underscoring the consistency and definition of attitudes within this population sample.

Table 2 provides a detailed analysis of attitudes toward suicide attempts, showing how the participants in the sample position themselves regarding various dimensions. The Prevention and Protectiondimension has a mean of 31.06, with a mode of 35 and a median of 33. This high mean score, along with negative skewness (Skewness = −2.44) and high kurtosis (Kurtosis = 7.34), suggests that most participants tend to highly value Prevention and Protection efforts in relation to suicide attempts. The *p*-value (<0.001) indicates a significant distribution, reflecting strong consistency in this attitude within the sample.

Regarding “Individual Help”, a mean of 18.66 is observed, with a mode of 21 and a median of 19. The negative skewness (Skewness = −2.42) and high kurtosis (Kurtosis = 9.20) highlight a similar trend to the previous dimension, where most participants value personalized help in the context of suicide attempts. The “Institutional Help” dimension records the highest mean (58.67), with a mode of 61 and a median of 60. This dimension also shows negative skewness (Skewness = −1.44), reflecting a significant inclination toward the importance of institutional intervention in suicide attempt situations.

The “Triggers and Psychopathology” and “Causal Attributions” dimensions have means of 38.32 and 29.67, respectively. Both show significant distributions with moderate negative skewness (Skewness = −1.311 and −0.652), suggesting a consistent perception of the importance of understanding the triggering and causal factors in suicide attempts. Finally, “Medical Help” has a mean of 26.02, with negative skewness (Skewness = −0.865), indicating that participants recognize the crucial role of medical support in managing suicide attempts. Overall, these results underscore a general tendency to value both individual and institutional support systems, emphasizing the importance of a multidimensional approach to the prevention and treatment of suicidal behavior.

Table 3 shows the scoring levels in attitudinal beliefs about suicidal behavior, divided into various dimensions. In the “Legitimization of Suicide” dimension, most participants are in the medium level (87.3%), with 12.4% in the high level and only 0.3% in the low level. This suggests that many individuals hold an intermediate position regarding the Legitimization of Suicide. For “Suicide in Terminally Ill Patients”, the trend is similar, with 85.6% in the medium level and 14.4% in the high level, indicating a nuanced understanding of suicide in terminal contexts.

In the “Moral Dimension of Suicide”, 74.3% of participants are in the medium level, while 13.8% are in the low level and 11.9% are in the high level, reflecting a diverse ethical perception of suicide. The “Own Suicide” dimension shows a significant majority in the medium level (89.5%) and 10.5% in the high level, suggesting that attitudes toward personal suicide are mostly moderate.

Regarding Attitudes Towards Suicide Attempts (ASETSA), the “Prevention and Protection” dimension has 89.5% of participants in the medium level and 10.5% in the low level, highlighting the importance attributed to these measures. “Individual Help” also shows a predominance of the medium level (89.0%), with 11.0% in the low level, suggesting a significant valuation of personalized help. In “Institutional Help”, 78.2% are in the medium level, while 13.0% are in the low level and 8.8% in the high level, reflecting a generally favorable but varied perception of institutions.

The “Triggers and Psychopathology” dimension shows 74.3% in the medium level and 13.6% in the high level, indicating a moderate to high understanding of these factors. “Causal Attributions” stands out with 64.7% of participants in the high level, suggesting a strong identification of causes attributed to suicidal behavior. Finally, in “Medical Help”, 76.0% are in the medium level, with 16.7% in the low level and 7.3% in the high level, showing a generally positive perception of medical assistance in these contexts.

Table 4 reveals that professional experience with suicidal patients has a significant impact on various dimensions of beliefs and attitudes toward suicidal behavior. Professionals with experience in treating these patients exhibit notably different attitudes regarding the Legitimization of Suicide (*p* = 0.032), although the effect size is small (0.144). Similarly, attitudes toward Suicide in Terminally Ill Patients show significant differences based on professional experience (*p* = 0.049), with a moderate effect size (0.132). The perception of institutional help stands out with a highly significant difference (*p* < 0.001) and a considerable effect size (0.231). Likewise, the understanding of triggers and the psychopathology of suicide also varies significantly with professional experience (*p* = 0.005), showing a moderate effect size (0.191). These findings highlight the influence of clinical experience in shaping professional attitudes toward suicide.

Table 5 explores the differences in beliefs and attitudes toward suicidal behavior based on non-professional experience with suicidal patients. The results indicate a general lack of significant differences in attitudes, with the Legitimization of Suicide showing a *p*-value of 0.913 and an effect size of 0.007. Attitudes toward Suicide in Terminally Ill patients and the Moral Dimension of Suicide also do not show significant differences (*p* = 0.359, effect size = −0.056; *p* = 0.628, effect size = 0.030). Although Individual Help shows a marginally significant *p*-value (*p* = 0.043) with an effect size of 0.124, most other dimensions, such as Prevention and Protection, Institutional Help, and Causal Attributions, do not present significant differences (*p* > 0.05). In conclusion, non-professional experience does not seem to significantly influence attitudes toward suicidal behavior.

For a more detailed analysis, a comparison of the evaluated dimensions of attitudes and beliefs toward suicide was conducted, taking into account the professional area.

Table 6 shows that in the dimension of Legitimization of Suicide, no significant differences were found between professional groups (*p* = 0.128), despite medical interns having a higher mean (M = 11.286) and greater variability in responses (SD = 6.397, coefficient of variation = 0.567) compared to other groups. Regarding Suicide in Terminally Ill Patients, significant differences were observed between groups (*p* = 0.029). Medical interns again showed a higher mean (M = 10.694) compared to other groups, suggesting greater acceptance of this practice within this specific group. However, the Moral Dimension of Suicide did not show significant differences between groups (*p* = 0.428), although physicians had a slightly higher mean (M = 15.654). As for the dimension of Own Suicide, no significant differences were found between professions (*p* = 0.245). These results indicate that, while there are some differences in perceptions of Suicide in Terminally Ill Patients, overall attitudes toward suicide are similar among the health professions analyzed.

To identify specific aspects of the findings, post hoc Dunn’s comparisons were conducted, revealing significant differences in the dimensions of the applied scales among the professions analyzed. Specifically, the comparison between medical and nursing interns revealed a significant difference with a z-value of 2.608 and a *p*-value of 0.009. However, this difference did not remain significant after Bonferroni correction (*p* = 0.091) and was marginally significant after Holm correction (*p* = 0.082). On the other hand, when comparing medical interns with nursing interns, a more pronounced significant difference was observed, with a z-value of 2.947 and a *p*-value of 0.003, which remained significant even after both corrections (*p* = 0.032). These findings suggest significant variations in beliefs and attitudes toward suicidal behavior between medical interns and other professional groups, although these differences tend to diminish when adjusting for multiple comparisons. In contrast, other group comparisons, such as between medicine and nursing assistants, and between nursing and nursing interns, did not show significant differences, indicating similarities in the responses of these groups.

Table 7 shows the analysis of attitudes toward suicide prevention, where significant differences were found in the Institutional Help dimension, with a *p*-value of 0.003. In this case, nurses had a higher mean (M = 60.727) compared to medical interns, who had the lowest mean (M = 54.327). Additionally, the Triggers and Psychopathology dimension also revealed significant differences between professions, with a *p*-value of 0.001. Medical interns obtained a lower mean (M = 34.469) than the other groups, indicating variations in the perception of factors influencing suicide.

The post hoc Dunn’s test between different professions for the factor termed “Institutional Help” identified significant differences in several comparisons. The comparison between medical interns and nurses yielded a z-value of −3.874, with a *p*-value of less than 0.001, indicating a statistically significant difference. Moreover, medical interns also showed significant differences with nursing assistants, with a z-value of −3.137 and a *p*-value of 0.002. These differences highlight the variability in perceptions and attitudes toward suicide and its prevention among the various professions studied. On the other hand, the comparison between physicians and medical interns showed a z-value of 2.351 and a *p*-value of 0.019, suggesting that perceptions may vary even within the same discipline between professionals and interns. These results underscore the importance of considering the professional context when addressing mental health and suicide prevention issues.

Regarding the “Triggers and Psychopathology” factor, which also showed significant differences, the post hoc Dunn’s analysis between different professions revealed significant differences in several cases. Medical interns showed a statistically significant difference compared to nursing interns, with a z-value of −3.290 and a *p*-value of 0.001, adjusted to 0.010 according to the Bonferroni correction and 0.008 with the Holm correction. Likewise, medical interns presented significant differences compared to nursing assistants, with a z-value of −4.314 and a *p*-value of less than 0.001, which remained significant after both Bonferroni and Holm corrections. These differences indicate notable variations in attitudes and perceptions toward suicide among professional groups, suggesting the need for personalized approaches in education and training on mental health topics.

## 4. Discussion

The objective of this research was to analyze the attitudes and beliefs regarding suicidal behavior and attempts among healthcare professionals. The results show that participants tend to exhibit medium to high scores in the Legitimization of Suicide, indicating a propensity to justify it, as well as favorable attitudes toward suicide in patients with terminal illnesses. This suggests that most participants view suicide as an option in situations of extreme suffering. These findings partially coincide with those of Yuncar-Fajardo et al., 2023 [5], who identified favorable attitudes toward Suicide in Terminally Ill Patients but rejected the Legitimization of Suicide.

While in the study by Yuncar-Fajardo et al., 2023, the sample focused on a specific population with cultural characteristics that may influence the perception of Suicide in Terminally Ill Patients, our study reflects a Colombian context with sociocultural and religious influences that could explain a greater acceptance of suicide in cases of extreme suffering, but without extending such legitimization to other contexts, such as suicide in young individuals or those without terminal illnesses. Similarly, the professional training and clinical experience of the participants in both studies may have influenced the variation in attitudes toward the Legitimization of Suicide [5,31].

Moreover, the observed differences in attitudes toward Suicide in Terminally Ill Patients may also be related to the perception of the role of healthcare professionals in decision-making regarding the end of life, an issue that may be more developed in certain professional contexts than in others. These aspects may explain why our results partially coincide, leading us to a more critical and nuanced conclusion about the need to consider both the cultural context and professional training when interpreting attitudes toward suicide [31,32].

This aspect is relevant, as the Legitimization of Suicide refers to the extent to which individuals consider the act of taking one’s own life acceptable or justifiable under certain circumstances. In this sense, high scores in this factor can be interpreted as the acceptance that suicide is a valid option under certain conditions, such as in elderly individuals, in situations of extreme suffering, or when the decision to commit suicide is perceived as a personal matter in which others should not intervene. These results differ from studies conducted with nursing professionals who interact with patients with mental illnesses and suicidal risk, who tend to disagree with the idea of legitimizing suicide, suggesting that, despite their continuous contact with individuals at suicidal risk, they do not develop understanding attitudes toward this behavior, firmly rejecting it [10,33].

In line with the previous findings and with previous research [34], the Moral Dimension of Suicide showed a strong moral perception, suggesting that most participants consider suicide from an ethical or moral perspective. High scores, as observed in the research subjects, suggest that suicide is perceived as an ethical and social transgression; it is seen as immoral, contrary to social norms, and an affront to society, considering it an act that should be prohibited and sanctioned, equating it with murder.

These results may reinforce the condemnation within a moral framework that values life as a supreme good that must be preserved and which may be linked to religious beliefs. In this sense, analyzing this dimension in different populations is necessary, as it seems to constitute a protective factor for the general population, but a risk factor in the care of patients with this behavior by healthcare professionals [5,35,36,37].

Regarding the perception of suicide itself, referring to a person’s predisposition to consider suicide as a viable option in response to extreme situations, loneliness, or depression, this dimension captures the individual’s internal struggle between absolute rejection of suicide and the perception that, in certain moments of despair or when life’s problems become insurmountable, suicide could be seen as the only escape. In this respect, the participants in this study showed a tendency toward low scores, which seems to differ from findings in other studies that have reported high levels of suicidal risk among healthcare professionals [38,39].

This study also explored attitudes toward suicide attempts among healthcare professionals, identifying that most rated the “Prevention and Protection” subscale at medium to low levels. These results indicate a limited adoption of a comprehensive and multidimensional approach to managing suicidal risk, which should include not only medical intervention but also community and family support. This approach is essential to prevent future attempts, as it allows healthcare professionals to broaden their perspective beyond the clinical setting, reaffirming that with proper treatment and support, it is possible to significantly reduce the risk. Additionally, the importance of the social reintegration of people who have attempted suicide is emphasized, highlighting that suicide should not be considered a solution [26,40].

Regarding the “Individual Help” dimension, it was observed that healthcare personnel predominantly showed medium scores, indicating a personal and emotional willingness to provide support and understanding to people who have attempted suicide, but which is not clearly defined. These findings reveal the urgent need to implement training strategies that emphasize the importance of recognizing the high risk to which these individuals are exposed and the critical need to offer them close and empathetic support. It is essential that healthcare personnel understand the urgency of providing emotional support and practical assistance to prevent future suicide attempts. The results of this study do not show a strong commitment from the participants to actively help these individuals, highlighting the need to raise awareness among healthcare personnel about the importance of direct and comprehensive action to support those who have attempted suicide [10,13,41]

On the other hand, the low scores observed in the dimensions of “Prevention and Protection”, “Individual Help”, “Institutional Help”, and “Medical Help” reflect significant concerns in the attitudes and perceptions of healthcare professionals toward patients who have attempted suicide. The low scores in these dimensions suggest that professionals perceive deficiencies in the treatment and care of these patients, which may be related to a lack of specific training in managing suicidal crises and an organizational culture that does not prioritize compassionate and humane care. This is concerning, as proper and empathetic treatment is essential for the recovery and well-being of patients who attempt suicide [42,43].

Contrary to what was previously stated, the “Triggers and Psychopathology” and “Causal Attributions” dimensions showed tendencies toward medium to high scores among the participants, indicating a moderate to solid understanding of the psychological and psychiatric causes that can lead to a suicide attempt. This suggests that healthcare professionals recognize the importance of mental health conditions and specific triggering factors, such as depressive disorders, anxiety, or traumatic events, in influencing suicidal behavior. Moreover, the high scores in “Causal Attributions” reflect a nuanced perception of the reasons behind suicide attempts, considering both individual factors and the social and environmental circumstances that may contribute to a person’s decision to take their own life. These findings highlight the need to maintain and strengthen the multidimensional understanding of suicide to improve interventions and the management of these complex cases [9].

Furthermore, the role of experience in treating suicidal patients was investigated, identifying that those with such experience exhibit significantly different attitudes toward the Legitimization of Suicide, although the observed effect size is small. Similarly, attitudes toward Suicide in Terminally Ill Patients show significant variations depending on professional experience, with a moderate effect size. Notably, the perception of institutional help presents highly significant differences with a considerable effect size. Additionally, understanding the triggers and psychopathology of suicide also varies significantly with professional experience, showing a moderate effect size. These findings underscore the influence of clinical experience in shaping healthcare professionals’ attitudes toward suicide, suggesting that ongoing practice in this field can shape more nuanced perspectives adapted to the complexities of suicidal behavior [44,45].

On the other hand, regarding differences in beliefs and attitudes toward suicidal behavior based on non-professional experience with suicidal patients, the results did not reveal significant differences. Consequently, non-professional experience does not seem to have a notable influence on attitudes toward suicidal behavior. This suggests that professional clinical training and experience play a more determinant role in shaping these attitudes compared to personal or non-professional experiences [46].

Regarding potential differences by professional area, data analysis revealed significant differences in attitudes toward suicidal behavior. In particular, it was observed that medical interns and nursing interns showed notable variations in their beliefs and attitudes. These differences are more pronounced when comparing medical interns with nursing assistants, suggesting a disparity in the perception of suicide between these groups. In contrast, no significant differences were found in comparisons between other professional groups, such as medicine and nursing assistants, or between nursing and nursing interns, indicating similarities in their responses [47].

Regarding attitudes toward suicide prevention, clear differences were identified in the perception of institutional help, with nurses showing a higher appreciation compared to medical interns. Additionally, the understanding of the triggers and psychopathology of suicide also varied between professions, with medical interns showing a lower perception of these factors compared to other groups. These differences underscore the need for educational approaches tailored to the professional context to improve mental health training and suicide prevention [10,48,49].

Additionally, the differences observed in attitudes among various healthcare professionals may be related to the nature of their contact with patients at risk of suicide. In Colombia, nurses, doctors, and nursing assistants do not always receive specific and sufficient training on how to effectively manage suicidal risk. This is reflected in the low scores in dimensions such as “Prevention and Protection” and “Individual Help”, suggesting a lack of preparedness to adopt comprehensive approaches that include not only medical intervention but also community and family support. This gap in training may lead professionals to underestimate the importance of prevention and early intervention in these cases.

Finally, it is important to highlight that professional experience in managing patients with suicide attempts also influences the attitudes of healthcare professionals in Colombia [50]. Those with more experience tend to show a deeper understanding of the triggering factors and psychopathological causes that lead a person to attempt suicide. However, the lack of significant differences in professionals’ attitudes based on non-professional personal experience underscores the need for clinical training and practice to be the primary determinants for developing comprehensive and effective attitudes toward managing suicidal risk in the Colombian context. The results obtained reveal important trends in the attitudes of healthcare professionals toward the care and prevention of suicide [4]. However, it is crucial to consider some limitations that affect the generalization of these findings (Mitchell, et al., 2020). The study focused on a specific geographic region, which may not represent the attitudes of all healthcare professionals in Colombia [28]. Additionally, the use of self-reported questionnaires may introduce social desirability bias, and the cross-sectional design of the study limits the ability to establish causal relationships between the analyzed variables. The absence of longitudinal follow-up also restricts the understanding of how attitudes may evolve over time and with professional experience [16].

However, the results of this study provide an enriched perspective on how attitudes and beliefs toward suicide vary among healthcare professionals in different contexts and roles, underscoring the need for continuous training programs that not only enhance technical knowledge but also address and modify attitudes and beliefs, especially in areas where stigma persists [21].

Future studies should be conducted with a longitudinal design to observe changes in attitudes and knowledge over time. Additionally, personalized educational interventions should be explored to address specific erroneous beliefs and stigmas, considering cultural and contextual differences. It would also be beneficial to investigate how institutional policies and organizational culture influence attitudes toward suicide and the management of complex cases [16,21,28].

## 5. Conclusions

The results reveal that a significant number of healthcare professionals consider suicide a legitimate option in cases of extreme suffering, such as in terminally ill patients. This suggests the need for critical reflection on the ethical and moral implications of these attitudes and how they may influence clinical care for these patients.

Medium to low scores were identified in the dimensions of Prevention and Protection, reflecting a limited adoption of a comprehensive and multidimensional approach to managing suicidal risk. This finding underscores the need to strengthen preventive strategies that include not only medical intervention but also community and family support.

Attitudes toward individual help and institutional help showed significant variability among different professional groups, especially between medical interns and other healthcare professionals. This indicates the importance of adapting training programs to the specific needs of each professional group to improve care for individuals at risk of suicide.

Professional experience in treating suicidal patients notably influences attitudes toward suicide, suggesting that continuous clinical practice can shape more nuanced and understanding attitudes. This highlights the need for ongoing training that addresses both technical knowledge and attitudes and beliefs.

The study emphasizes the importance of implementing institutional policies that promote more compassionate and humane care for patients at risk of suicide. Additionally, the need for continuous training programs that not only improve the technical competencies of healthcare professionals but also address erroneous beliefs and stigma related to suicide is highlighted.

## Figures and Tables

**Table 1:** Descriptive statistics of Attitudinal Beliefs about Suicidal Behavior (CCS-18).

	n	Mode	Median	M	IC 95% Upper	IC 95% Lower	σ	Skewness	Kurtosis	*p*-Value	Min	Max
Legitimization of Suicide	354	6.00	8.00	9.96	10.48	9.45	4.92	1.67	3.43	<0.001	1	35
Suicide in Terminally Ill Patients	354	4.00	6.50	8.68	9.29	8.07	5.86	1.50	1.50	<0.001	4	28
Moral Dimension of Suicide	354	10.00	14.00	14.66	15.35	13.98	6.57	0.22	−0.81	<0.001	4	28
Own Suicide	354	4.00	8.00	7.83	8.23	7.44	3.74	0.82	0.42	<0.001	4	23

n (sample), M (mean); σ (standard deviation); *p*-value (Shapiro–Wilk).

**Table 2:** Descriptive statistics of Attitudes Toward Suicide Attempts (ASETSA).

	n	Mode	Median	M	IC 95% Upper	IC 95% Lower	σ	Skewness	Kurtosis	*p*- Value	Min	Max
Prevention and Protection	354	35.00	33.00	31.06	31.63	30.49	5.43	−2.44	7.34	<0.001	5	35
Individual Help	354	21.00	19.00	18.66	18.96	18.36	2.85	−2.42	9.20	<0.001	3	21
Institutional Help	354	61.00	60.00	58.67	59.72	57.62	10.05	−1.44	4.16	<0.001	11	77
Triggers and Psychopathology	354	37.00	39.00	38.31	39.057	37.582	7.056	−1.311	3.294	<0.001	7	49
Causal Attributions	354	32.00	30.50	29.66	30.450	28.883	7.495	−0.652	0.341	<0.001	6	42
Medical Help	354	29.00	27.00	26.02	26.644	25.396	5.969	−0.865	0.826	<0.001	5	35

n (sample), M (mean); σ (standard deviation); *p*-value (Shapiro–Wilk).

**Table 3:** Scoring levels in the dimensions of Attitudinal Beliefs about Suicidal Behavior and Attitudes Towards Suicide Attempts.

Factor/Level CCS-18		*F*	%
Legitimization of Suicide	Low	1	0.3
	Medium	309	87.3
	High	44	12.4
Suicide in Terminally Patients	Low	303	85.6
	Medium	51	14.4
Moral Dimension of Suicide	High	49	13.8
	Medium	263	74.3
	High	42	11.9
Own Suicide	Medium	317	89.5
	High	37	10.5
Factor/Level ASETSA		*F*	%
Prevention and Protection	Low	37	10.5
	Medium	317	89.5
Individual Help	Low	39	11.0
	Medium	315	89.0
Institutional Help	Low	46	13.0
	Medium	277	78.2
	High	31	8.8
Triggers and Psychopathology	Low	43	12.1
	Medium	263	74.3
	High	48	13.6
Causal Attributions	Low	58	16.4
	Medium	67	18.9
	High	229	64.7
Medical Help	Low	59	16.7
	Medium	269	76.0
	High	26	7.3

*F* (frequency); % (percentage).

**Table 4:** Differences in Attitudinal Beliefs about Suicidal Behavior and Attitudes Towards Suicide Attempts according to professional experience with suicidal patients.

	U	*p*	Effect Size	Test of Equality of Variances (Brown-Forsythe)			
				F	df1	df2	*p*
Legitimization of Suicide	14,437.50	0.032	0.144	3.682	1	352	0.056
Suicide in Terminally Ill Patients	14,293.50	0.049	0.132	3.995	1	352	0.046
Moral Dimension of Suicide	12,443.00	0.836	−0.014	0.443	1	352	0.506
Own Suicide	15,052.00	0.004	0.192	1.976	1	352	0.161
Prevention and Protection	12,545.00	0.928	−0.006	0.130	1	352	0.719
Individual Help	13,917.50	0.125	0.103	2.688	1	352	0.102
Institutional Help	15,542.50	<0.001	0.231	4.196	1	352	0.041
Triggers and Psychopathology	15,032.50	0.005	0.191	5.321	1	352	0.022
Causal Attributions	14,418.50	0.038	0.142	0.514	1	352	0.474
Medical Help	13,717.50	0.205	0.087	3.613	1	352	0.058

U (Mann–Whitney U); *p* (*p*-value); F (F statistic value from an ANOVA); df1 and df2 (degrees of freedom associated with the ANOVA); *p* (*p*-value associated with the F test). Note: The effect size is given by the rank–biserial correlation.

**Table 5:** Differences in Attitudinal Beliefs about Suicidal Behavior and Attitudes Towards Suicide Attempts according to non-professional experience with suicidal patients.

	U	*p*	Effect Size	Test of Equality of Variances (Brown-Forsythe)			
				F	df1	df2	*p*
Legitimization of Suicide	15,286.00	0.913	0.007	0.176	1	352	0.675
Suicide in Terminally Ill Patients	14,329.50	0.359	−0.056	0.004	1	352	0.948
Moral Dimension of Suicide	15,643.00	0.628	0.030	0.235	1	352	0.628
Own Suicide	15,039.50	0.877	−0.010	0.137	1	352	0.712
Prevention and Protection	15,639.50	0.625	0.030	1.283	1	352	0.258
Individual Help	17,059.50	0.043	0.124	0.302	1	352	0.583
Institutional Help	14,449.00	0.438	−0.048	1.020	1	352	0.313
Triggers and Psychopathology	14,592.50	0.532	−0.039	0.494	1	352	0.483
Causal Attributions	15,503.50	0.736	0.021	2.814	1	352	0.094
Medical Help	14,020.50	0.219	−0.077	0.539	1	352	0.464

U (Mann–Whitney U); *p* (*p*-value); F (F statistic value from an ANOVA); df1 and df2 (degrees of freedom associated with the ANOVA); *p* (*p*-value associated with the F test). Note: The effect size is given by the rank–biserial correlation.

**Table 6:** Descriptive analysis and differences in Attitudinal Beliefs about Suicidal Behavior by professional area.

Factor	Profession	n	M	SD	Coefficient of Variation	k	df	*p*
Legitimization of Suicide	Nursing Assistant	113	10.07	4.60	0.45	7.151	4	0.128
	Nursing	88	9.94	4.81	0.48			
	Nursing Interns	26	8.61	4.59	0.53			
	Medical Interns	49	11.28	6.39	0.56			
	Medicine	78	9.46	4.48	0.47			
Suicide in Terminally Ill Patients	Nursing Assistant	113	8.29	5.22	0.63	10.812	4	0.029
	Nursing	88	7.97	5.19	0.65			
	Nursing Interns	26	7.15	5.71	0.79			
	Medical Interns	49	10.69	6.74	0.63			
	Medicine	78	9.29	6.62	0.71			
Moral Dimension of Suicide	Nursing Assistant	113	14.11	6.38	0.45	3.839	4	0.428
	Nursing	88	14.63	6.73	0.46			
	Nursing Interns	26	13.53	7.11	0.52			
	Medical Interns	49	15.04	5.85	0.38			
	Medicine	78	15.65	6.92	0.44			
Own Suicide	Nursing Assistant	113	8.07	3.72	0.46	5.446	4	0.245
	Nursing	88	8.21	3.54	0.43			
	Nursing Interns	26	7.26	4.23	0.58			
	Medical Interns	49	7.34	4.21	0.57			
	Medicine	78	7.57	3.51	0.46			

Note: n (number of subjects), M (mean), SD (standard deviation), k (Kruskal–Wallis), df (degrees of freedom), *p* (*p*-value).

**Table 7:** Descriptive analysis and differences in Attitudes Towards Suicide Attempts by professional area.

Factor	Profession	n	M	SD	Coefficient of Variation	k	df	*p*
Prevention and Protection	Nursing Assistant	113	31.02	5.04	0.16	2.052	4	0.726
	Nursing	88	30.72	5.94	0.19			
	Nursing Interns	26	31.23	6.60	0.21			
	Medical Interns	49	31.06	5.49	0.17			
	Medicine	78	31.46	5.03	0.16			
Individual Help	Nursing Interns	113	18.44	2.86	0.15	7.154	4	0.128
	Nursing	88	18.86	2.42	0.12			
	Nursing Interns	26	19.23	3.69	0.19			
	Medical Interns	49	18.16	3.31	0.18			
	Medicine	78	18.88	2.68	0.14			
Institutional Help	Nursing Assistant	113	59.07	10.04	0.17	16.161	4	0.003
	Nursing	88	60.72	8.56	0.14			
	Nursing Interns	26	58.80	13.13	0.22			
	Medical Interns	49	54.32	10.60	0.19			
	Medicine	78	58.46	9.52	0.16			
Triggers and Psychopathology	Nursing Assistant	113	39.12	7.16	0.18	23.386	4	0.001
	Nursing	88	39.47	5.86	0.14			
	Nursing Interns	26	38.65	9.41	0.24			
	Medical Interns	49	34.46	7.37	0.21			
	Medicine	78	38.15	6.33	0.16			
Causal Attributions	Nursing Assistant	113	29.08	7.74	0.26	0.980	4	0.913
	Nursing	88	30.42	6.96	0.22			
	Nursing Interns	26	29.07	9.08	0.31			
	Medical Interns	49	29.30	7.90	0.27			
	Medicine	78	30.09	6.95	0.23			
Medical Help	Nursing Assistant	113	25.29	5.47	0.21	6.157	4	0.188
	Nursing	88	26.62	5.81	0.21			
	Nursing Interns	26	26.92	7.56	0.28			
	Medical Interns	49	25.34	6.98	0.27			
	Medicine	78	26.51	5.54	0.20			

Note: n (number of subjects), M (mean), SD (standard deviation), k (Kruskal–Wallis), df (degrees of freedom), *p* (*p*-value).

## Data Availability

The datasets used and analyzed during the current study will be available from the corresponding author on reasonable request.
